# Multiple RNA virus matrix proteins interact with SLD5 to manipulate host cell cycle

**DOI:** 10.1099/jgv.0.001697

**Published:** 2021-12-09

**Authors:** Li Zhu, Xinyu Li, Henan Xu, Lifeng Fu, George Fu Gao, Wenjun Liu, Linqing Zhao, Xiaojun Wang, Wei Jiang, Min Fang

**Affiliations:** ^1^​ CAS Key Laboratory of Pathogenic Microbiology and Immunology, Institute of Microbiology, Chinese Academy of Sciences, Beijing 100101, PR China; ^2^​ State Key Laboratory of Veterinary Biotechnology, Harbin Veterinary Research Institute, The Chinese Academy of Agricultural Sciences, Harbin 150069, PR China; ^3^​ University of Chinese Academy of Sciences, Beijing 100049, PR China; ^4^​ Laboratory of Virology, Beijing Key Laboratory of Etiology of Viral Diseases in Children, Capital Institute of Pediatrics, Beijing 100020, PR China; ^5^​ International College, University of Chinese Academy of Sciences, Beijing 100049, PR China

**Keywords:** SLD5, RNA viruses, M protein, cell cycle, viral replication

## Abstract

The matrix protein of many enveloped RNA viruses regulates multiple stages of viral life cycle and has the characteristics of nucleocytoplasmic shuttling. We have previously demonstrated that matrix protein 1 (M1) of an RNA virus, influenza virus, blocks host cell cycle progression by interacting with SLD5, a member of the GINS complex, which is required for normal cell cycle progression. In this study, we found that M protein of several other RNA viruses, including VSV, SeV and HIV, interacted with SLD5. Furthermore, VSV/SeV infection and M protein of VSV/SeV/HIV induced cell cycle arrest at G0/G1 phase. Importantly, overexpression of SLD5 partially rescued the cell cycle arrest by VSV/SeV infection and VSV M protein. In addition, SLD5 suppressed VSV replication *in vitro* and *in vivo*, and enhanced type Ⅰ interferon signalling. Taken together, our results suggest that targeting SLD5 by M protein might be a common strategy used by multiple enveloped RNA viruses to block host cell cycle. Our findings provide new mechanistic insights for virus to manipulate cell cycle progression by hijacking host replication factor SLD5 during infection.

## Introduction

M protein is an essential component of many enveloped viruses. As major structural proteins, they usually form a single matrix layer underlying the envelope to mediate interactions between the envelope and the viral ribonucleoproteins [1, 2]. M protein also exerts multiple functions during infection, such as regulating viral transcription, virus assembly, budding and the morphology of viral particle [2]. M protein from Influenza A virus, vesicular stomatitis virus (VSV) and measles virus (MeV) has intrinsic budding propensity, as they are able to promote the virus-like particles (VLPs), budding when expressed alone in the absence of other viral components [3–5] . Retroviral M proteins are fatty acylated, allowing them to interact with cell membrane to form the budding site [6]. VSV M protein is responsible for condensing the viral nucleocapsid into a tightly coiled helix, giving the virus a bullet-like shape [7]. For the members of *Filoviridae* family, M proteins (VP40) play a role in the maintenance of filamentous morphology [8, 9]. In addition, most matrix proteins can shuttle into and out of the nucleus during infection. For example, influenza virus replicates in the nucleus, M1 enters the nucleus to help the viral ribonucleoproteins nuclear export after being synthesized in the cytoplasm [10]. The M proteins of other RNA viruses, such as Sendai virus (SeV) and VSV, have also been observed in the nucleus, although the replication of those viruses occur exclusively in the cytoplasm [11, 12]. However, their function in the nucleus and their interaction with host factors are currently not very clear.

Viruses depend on host cell resources to replicate their genome and have evolved multiple strategies to manipulate cell cycle progression in order to provide favourable conditions for their own replication [13]. Influenza A virus and its nonstructural protein NS1 have been shown to block host cell cycle in the G0/G1 phase [14, 15]. Recently, we demonstrated that M1 of influenza virus is also responsible for the cell cycle arrest [16]. Cell cycle arrest can be found in many viral infections. For example, some small DNA viruses, such as human papillomavirus, adenoviruses and simian virus 40, lack their own polymerase and rely on host cell DNA replication machinery for replication of viral genomes, so they must promote cell entry into the S phase [17, 18]. In contrast, herpesviruses are extremely successful DNA viruses that encode their own DNA polymerase and accessory factors and are able to elicit cell cycle arrest in the G1/S interface so that host DNA replication is blocked [19]. Besides DNA virus, many RNA viruses induce cell cycle arrest in G1, S or G2 phase to favour viral replication, respectively. The nonstructural proteins 3b and 7a of severe acute respiratory syndrome coronavirus (SARS-CoV), respiratory syncytial virus (RSV) and its M protein all induce cell cycle arrest at the G0/G1 phase [20–23]. Enterovirus 71 (EV71) mediates cell cycle arrest in S phase via nonstructural protein 3D [24]. Human immunodeficiency virus (HIV) type-1 Vif triggers an accumulation of infected cells in the G2 phase [25]. Cell cycle arrest may inhibit early death of infected cells, allowing cells to escape immune defence or promoting viral assembly [14, 26]. There are increasing evidences showing that viral infection or expression of certain viral proteins regulate the progress of host cell cycle. However, the effect of more RNA viruses on host cell cycle and the molecular mechanisms behind these phenomena need further investigations.

SLD5 is a component of the GINS complex, which is essential for both the initiation and elongation stages of the replication process. CDC45 associates with MCM and GINS to form the CMG complex with helicase activity that unwinds the duplex DNA ahead of the moving replication fork [27, 28]. In drosophila, SLD5 is necessary for normal cell cycle progression and maintenance of genomic integrity [29]. Attenuation of SLD5 expression blocks cell cycle at the G0/G1 phase [16, 30]. So far, studies of SLD5 mainly focus on its role in DNA replication and few studies have linked it to viral infections. For virus infection, we recently discovered that SLD5 inhibits influenza virus replication and is a target of influenza virus M1 to regulate host cell cycle [16].

In this study, we demonstrated that M proteins from multiple RNA viruses, such as SeV, VSV, ZEBOV and HIV, all interacted with SLD5, suggesting SLD5 might be an important target for RNA virus to manipulate host cell cycle.

## Results

### M protein from multiple RNA viruses interacts with SLD5

**Fig. 1.**
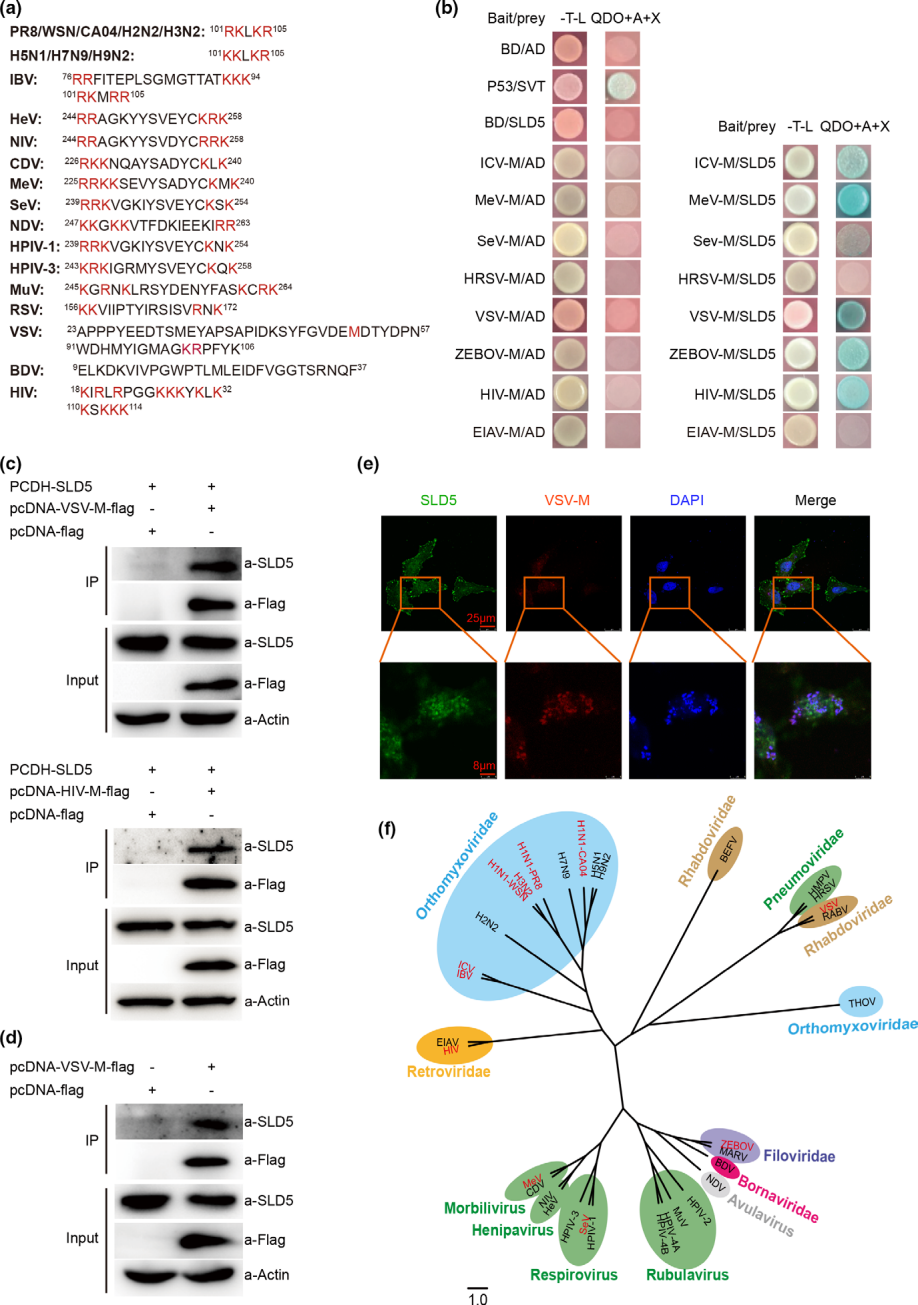
SLD5 interacts with matrix proteins of several RNA viruses. (a) The nuclear location sequences of M proteins of different RNA viruses were listed. Critical amino acids contributing to a NLS motif are coloured red. (b) Interaction between SLD5 and M proteins by the yeast two-hybrid assay. SLD5 was fused to the pGADT7 vector, M from ICV, MeV, SeV, VSV, ZEBOV, HIV and EIAV were fused to the pGBKT7 vector. The indicated plasmids were co-transformed into yeast strain Y2HGold. Transformants were selected for growth on -T-L medum. The colonies were then transferred to -T-L (left) and QDO+A+X (right) plates. (c) Co-immunoprecipitation between SLD5 and VSV-M-flag/HIV-M-flag. In total, 293 T cells were co-transfected with PCDH-SLD5 and VSV-M-flag/HIV-M-flag plasmids or the empty vector pcDNA3.0-flag. Then, 36 h post-transfection, the cells lysates were immunoprecipitated (IP) with anti-flag affinity gel and then subjected to immunoblotting (IB) with the indicated antibodies. (d) Co-immunoprecipitation between endogenous SLD5 and VSV-M-flag. Lysates of 293 T cells transfected with the pcDNA3.0-flag or pcDNA3.0-VSV-M-flag were IP with anti-flag affinity gel, followed by IB with the indicated antibodies. (e) Co-localization of SLD5 and VSV M. HeLa cells were co-transfected with pcDNA4.0-SLD5-HA and pcDNA3.0-VSV-M-flag plasmids, 24 h later, cells were fixed and stained with anti-flag and anti-HA antibodies, followed by fluorescein isothiocyanate (FITC)- and tetramethylrhodamine (TRITC)-conjugated secondary antibodies, then analysed by confocal microscopy. Representative micrographs with scale bars representing 8 µm and 25 µm. (f) Unrooted phylogenetic tree of selected RNA viruses based on the M proteins. Complete M amino acid sequences were aligned by ClustalW using mega 7 and analysed by the maximum-likelihood method. The cladogram was generated using Fig Tree v1.4.4. Scales bars indicate amino acid substitutions per site. The tree is overlaid with coloured ellipses representing the various virus families or genera.

Many RNA viruses, including members from *Orthomyxoviridae, Paramyxoviridae, Rhabdoviridae, Filoviridae, Bornaviridae* and *Retroviridae* families, have an M protein located inside the viral envelope (Table 1). These M proteins exhibit some similar characteristics, such as mediating the association between viral envelope and core, regulating virus assembly and budding. In addition, almost all M proteins from these viruses contain nuclear localization sequences (NLSs) and can enter the nucleus (Table 1, Fig. 1a). We previously demonstrated influenza virus M1 interacts with SLD5 [16]. To investigate whether M protein of other viruses also interacts with SLD5, we firstly used a yeast two-hybrid assay. The full-length M coding sequence of ICV, MeV, SeV, HRSV, VSV, ZEBOV, HIV and EIAV were cloned into the pGBKT7 plasmid to generate pGBKT7-M as a bait, the bait construct was co-transformed into the yeast strain Y2H Gold with the prey plasmid pGADT7-SLD5, followed by screening on Trp-/Leu- (-T-L) and high stringency plates (SD/-Ade/-His/-Leu/-Trp/+AbA/+X-α-Gal, QDO+A+X). Surprisingly, six viral M proteins out of the total of eight tested, all interacted with SLD5, including M proteins from ICV, MeV, SeV, VSV, ZEBOV and HIV. Meanwhile, M protein of HRSV and EIAV showed no interaction with SLD5 in this assay (Fig. 1b). Interestingly, RSV M2-1 protein, a transcriptional processivity and anti-termination factor, which is found in the nuclei of M2-1 expressing cells and in RSV-infected cells [31], showed interaction with SLD5 in our yeast two-hybrid assay (Fig. S1, available in the online verstion of this article).

**Table 1.** Most M proteins from enveloped RNA viruses contain NLS

Family/Subfamily	Genus	Virus name	Genome type	GeneBank accession no.	Length of M	NLS reported
**Orthomyxoviridae**	Influenza A virus	A/Puerto Rico/8/1934, H1N1-PR8	Eight segments, ssRNA, -	NP_040978.1	252aa	[52]
		A/WSN/1933, H1N1-WSN	Eight segments, ssRNA, -	AAA43252.1	252aa	[53–56]
		A/California/04/2009, H1N1-CA04	Eight segments, ssRNA, -	ANE02047.1	252aa	[54]
		A/Korea/426/1968, H2N2	Eight segments, ssRNA, -	YP_308854.1	252aa	–
		A/New York/392/2004, H3N2	Eight segments, ssRNA, -	YP_308841.1	252aa	[57]
		A/goose/Guangdong/1/1996, H5N1	Eight segments, ssRNA, -	YP_308671.1	252aa	[57]
		A/Anhui/1-YK_RG03/2013, H7N9	Eight segments, ssRNA, -	AKU41056.1	252aa	[58]
		A/chicken/Shandong/lx929/2007, H9N2	Eight segments, ssRNA, -	AEB71223.1	252aa	[59]
	Influenza B virus	B/Lee/1940, IBV	Eight segments, ssRNA, -	NP_056664.1	248aa	[60, 61]
	Influenza C virus	C/Ann Arbour/1/50, ICV	Seven segments, ssRNA, -	YP_089657.1	242aa	–
	Thogotovirus	Thogoto virus, THOV	Six segments, ssRNA, -	YP_145806.1	266aa	[62]
**Paramyxiviridae**						
Paramyxivirinae	Avulavirus Henipavirus	Newcastle disease virus, NDV	Nonsegmented, ssRNA, -	YP_009513196.1	364aa	[63–65]
	Henipavirus	Hendra virus, HeV	Nonsegmented, ssRNA, -	NP_047110.2	352aa	[66–68]
		Nipah virus, NIV	Nonsegmented, ssRNA, -	NP_112025.1	352aa	[67–69]
	Morbilivirus	Canine distemper virus, CDV	Nonsegmented, ssRNA, -	NP_047204.1	335aa	[69, 70]
		Measles virus, MeV	Nonsegmented, ssRNA, -	NP_056921.1	335aa	[67, 71]
	Respirovirus	Human parainfluenza virus 1, HPIV-1	Nonsegmented, ssRNA, -	NP_604439.1	348aa	–
		Human parainfluenza virus 3, HPIV-3	Nonsegmented, ssRNA, -	NP_067150.1	353aa	–
		Sendai virus, SeV	Nonsegmented, ssRNA, -	NP_056876.1	348aa	[11, 67, 70]
	Rubulavirus	Human parainfluenza virus 2, HPIV-2	Nonsegmented, ssRNA, -	NP_598403.1	377aa	–
		Human parainfluenza virus 4A, HPIV-4A	Nonsegmented, ssRNA, -	YP_008378662.1	382aa	–
		Human parainfluenza virus 4B, HPIV-4B	Nonsegmented, ssRNA, -	AFB82775.1	382aa	–
		Mumps virus, MuV	Nonsegmented, ssRNA, -	NP_054710.1	375aa	[36]
Pneumovirinae	Metapneumovirus	Human metapneumovirus, HMPV	Nonsegmented, ssRNA, -	YP_009513267.1	254aa	[72]
	Pneumovirus	Human respiratory syncytial virus; HRSV	Nonsegmented, ssRNA, -	API65185.1	256aa	[73–75]
**Rhabdoviridae**	Vesiculovirus	Vesicular stomatitis Indiana virus, VSV	Nonsegmented, ssRNA, -	NP_041714.1	229aa	[36]
	Lyssavirus	Rabies virus, RABV	Nonsegmented, ssRNA, -	NP_056795.1	202aa	[76, 77]
	Ephemerovirus	Bovine ephemeral fever virus, BEFV	Nonsegmented, ssRNA, -	NP_065401.1	223aa	[78]
**Filoviridae**	Ebolavirus	Zaire ebolavirus, ZEBOV	Nonsegmented, ssRNA, -	NP_066245.1	326aa	[79, 80]
	Marburgvirus	Marburg marburgvirus, MARV	Nonsegmented, ssRNA, -	YP_001531155.1	303aa	[81, 79]
**Bornaviridae**	Bornavirus	Borna disease virus 1, BDV	Nonsegmented, ssRNA, -	NP_042022.1	142aa	[82]
**Retroviridae**	Lentivirus	Human immunodeficiency virus-1, HIV	Two identical strands, ssRNA, +	NP_579876.2	132aa	[83, 84]
		Equine Infectious Anaemia Virus, EIAV	Nonsegmented, ssRNA, +	NP_056901.1	124aa	–

–, not reported.

To further confirm the SLD5-M interaction, we chose M of VSV and HIV for co-immunoprecipitation experiments. SLD5 was co-transfected with flag-tagged M or the empty vector in 293 T cells. As shown in Fig. 1(c), the interaction between VSV/HIV M and SLD5 were further confirmed. To investigate whether VSV M interacts with endogenous SLD5, 293 T cells were transfected with the pcDNA3.0-flag or pcDNA3.0-VSV-M-flag, as shown in Fig. 1(d), VSV M specifically immunoprecipitated cellular SLD5. We further examined whether SLD5 and VSV M co-localize inside cells using confocal microscopy. SLD5 and M proteins were widely distributed in cytoplasm and nucleus, and showed co-localization (Fig. 1e).

Our above results indicated that M protein from multiple RNA viruses interacts with SLD5. We then compared the amino acid sequences of the M protein from the RNA viruses listed in Table 1, part of the sequence alignment results was shown in Fig. S2, the sequences of M protein were not conserved. In addition, the binding sites of SLD5 and M of PR8/VSV/ZEBOV/HIV were predicted by discovery studio 2.5. As shown in Fig. S3, all these M proteins have multiple interacting sites with SLD5, but their binding sites to SLD5 varied with different M, which might be due to the large differences in the structure and sequence of the M proteins. Phylogenetic analysis of the M proteins revealed that the genetic distance of viruses from the same family/subfamily or genus is closer, except for the THOV, although it belongs to the *Orthomyxoviridae* family, it is far away from other family members and form a monophyletic clade. The viruses were labelled with red letters with their M proteins tested positive in the yeast two-hybrid assay, showing that viruses with M protein interacting with SLD5 are distributed among various families (Fig. 1f).

### VSV/SEV infection arrests host cell cycle at G0/G1 phase

**Fig. 2.**
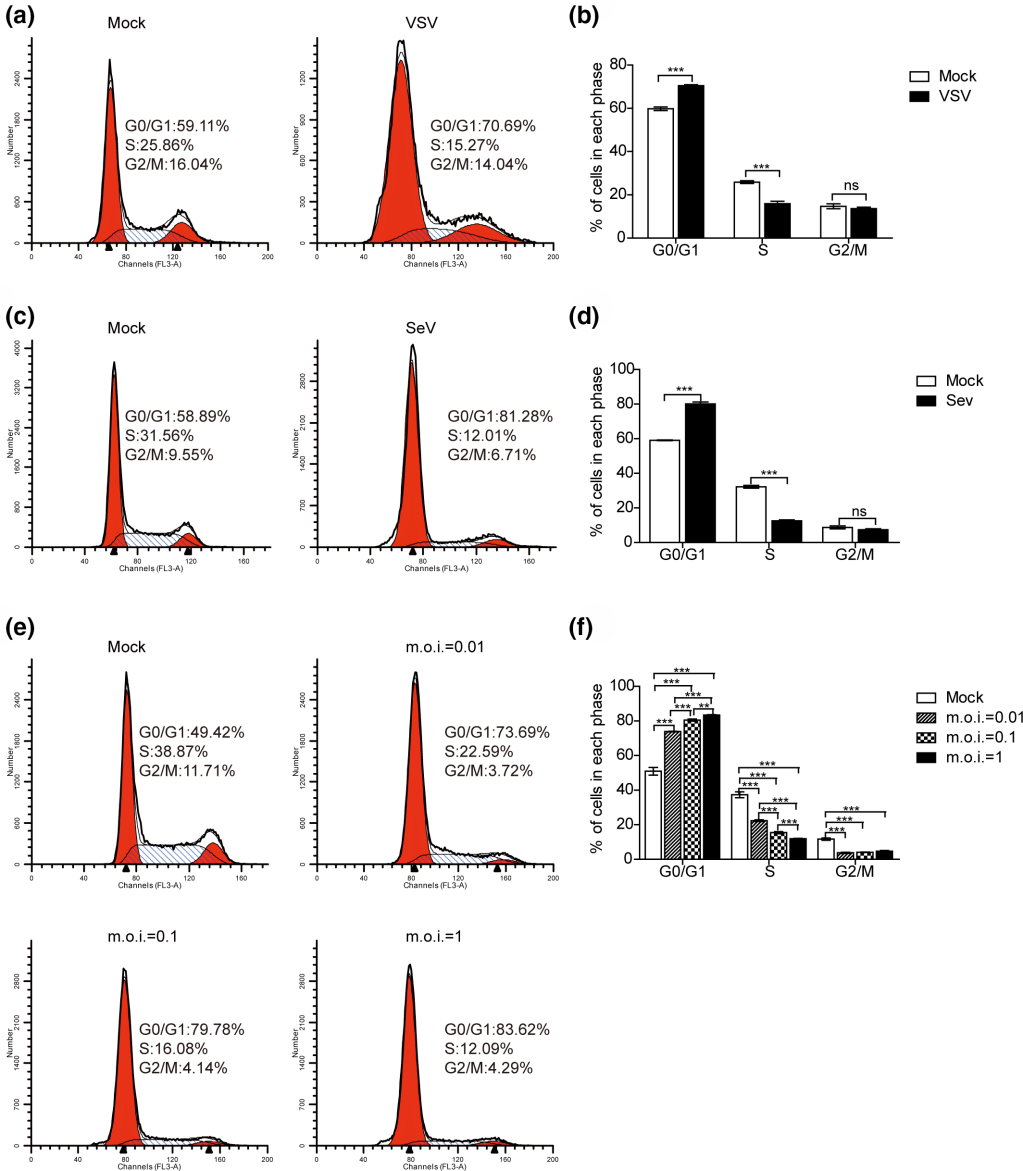
VSV and SeV infection induce cell cycle arrest at G0/G1 phase. HeLa cells were mock infected or infected with VSV-GFP (a) and SeV (c) at an m.o.i. of 1, 24 h later, cells were collected for analysing cell cycle profiles by flow cytometry. (b, d) The histograms displaying the cell cycle distribution were analysed by the ModFit LT programme, VSV-GFP (b), SeV (d). (e) A549 cells were synchronized at G0/G1 phase by serum starvation for 48 h and then mock infected or infected with VSV-GFP at the indicated m.o.i. Then, 18 h later, cells were collected for analysing cell cycle profiles by flow cytometry. (f) The percentage of cells in each phase of the cell cycle is shown. Data correspond to the mean±sd of at least three independent experiments. **P<0.01, ***P<0.001.

To investigate the impact of the infection of more RNA viruses on host cell cycle progression, we used flow cytometry analysis to assess the effect of virus infection on cell cycle. Firstly, HeLa cells were infected with VSV at a m.o.i. of 1. The cells were collected and analysed at 24 h post-infection (h p.i.). Nuclear DNA contents were measured by using propidium iodide (PI) staining and PI positive cells were analysed, the gating strategies are shown in Fig. S4a. VSV infection resulted in a significant accumulation of cells in the G0/G1 phase (as 70.41±0.69% in VSV-infected cells compared to 59.80±1.14% in mock-infected cells) (Fig. 2a, b). Secondly, the effect of SeV infection on HeLa cells cycle was also analysed. As shown in Fig. 2(c, d), SeV infection also significantly altered the cell cycle profile and increased the proportion of cells in G0/G1 phase. To further determine the effects of VSV infection on host cell cycle, A549 cells were firstly synchronized in the G0/G1 phase by serum starvation for 48 h and then mock infected or infected with the virus at different m.o.i. As shown in Fig. 2(e, f), VSV infection in A549 cells also resulted in host cell cycle arrested at the G0/G1 phase, the higher dose of VSV infection, the more cells accumulated in the G0/G1 phase. Thus, VSV and SeV infection induced host cell cycle arrested at G0/G1 phase.

### SLD5 partially rescues VSV/SeV infection-induced host cell G0/G1 arrest

**Fig. 3.**
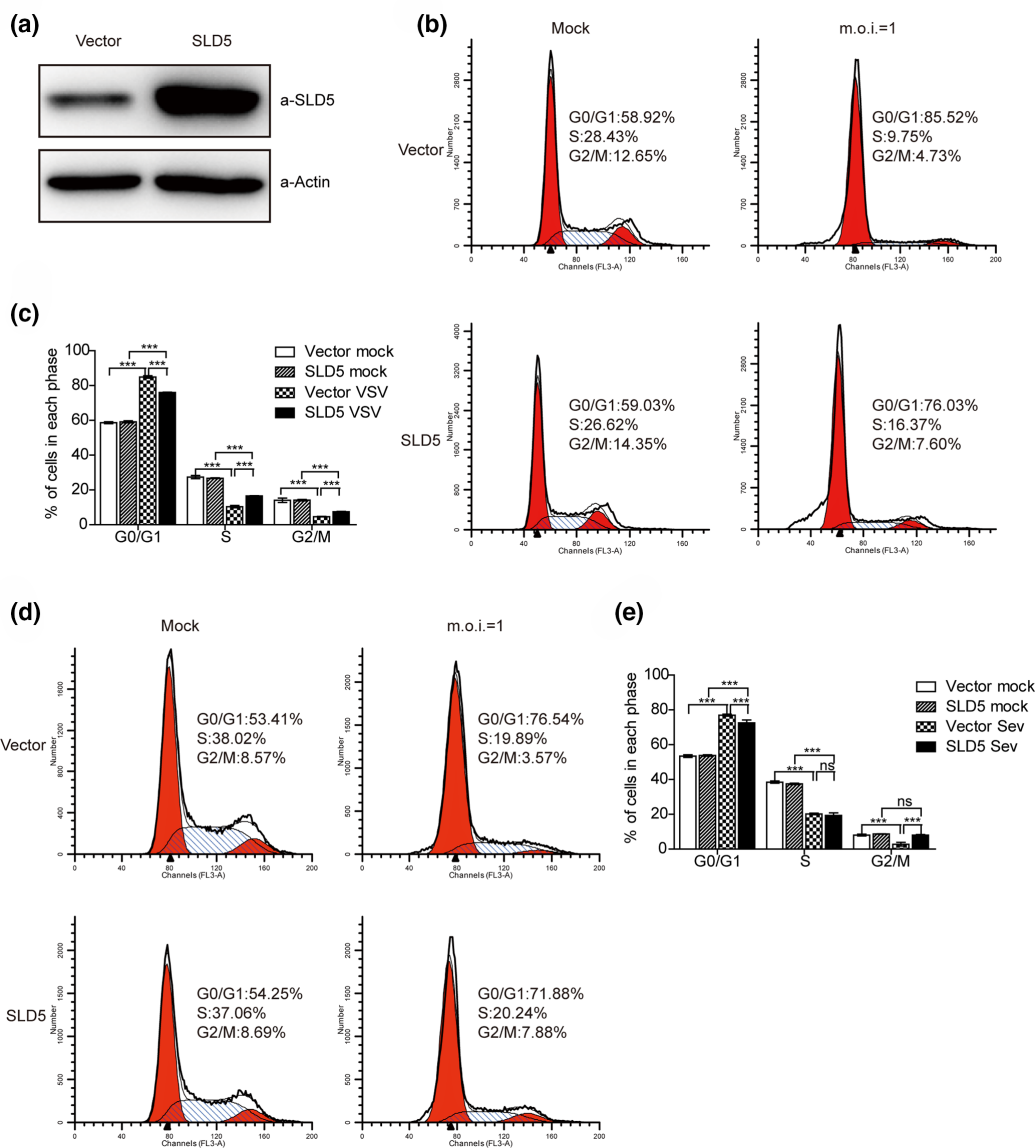
SLD5 partially rescues cell cycle arrest induced by VSV and SeV infection. (a) Western blot for detection of SLD5 expression level in A549 SLD5 overexpression or control cells transduced with the indicated lentiviral systems. (b, d) Cell cycle of A549 SLD5 overexpressing and control cells after VSV-GFP (b) and SeV (d) infection. A549 cells stably expressing SLD5 or control cells were serum-starved for 48 h, and then mock infected or infected with VSV-GFP and SeV at an m.o.i. of 1. Then, 18 h later, cells were collected and cell cycle profiles were determined by flow cytometry. (c, e) The percentage of cells in each phase of the cell cycle is shown, VSV-GFP (c), SeV (e). Data correspond to the mean±sd of at least two independent experiments. **P<0.01, ***P<0.001.

Our above data have shown that M protein from VSV or SeV interacted with SLD5, and VSV/SeV infection induced host cell cycle arrested at G0/G1 phase, suggesting that M-SLD5 interaction might contribute to the infection-induced cell cycle arrest. If this is true, overexpression of SLD5 should rescue cell cycle arrest resulted by VSV/SeV infection. Thus, we generated a SLD5-overexpressing A549 cell line, and the increased SLD5 expression level was confirmed by Western blot (Fig. 3a). The SLD5 overexpressing A549 cells and control cells were infected with VSV and collected at 18 h p.i. to analyse cell cycle. As shown in Fig. 3(b, c), there were no differences in the cell cycle between the control cells and SLD5 overexpressing cells in the absence of virus infection. However, overexpression of SLD5 significantly rescued the G0/G1 phase arrest after VSV infection (as 75.92±0.42% in SLD5 overexpressing cells compared with 85.90±0.97% in control cells). We further infected SLD5 overexpressing cells and the control cells with SeV, and got similar results as VSV infection (Fig. 3d, e). Thus, SLD5 partially rescued VSV/SeV infection-induced G0/G1 arrest.

### M protein from VSV, SeV and HIV induces host cell cycle arrest in the G0/G1 phase

**Fig. 4.**
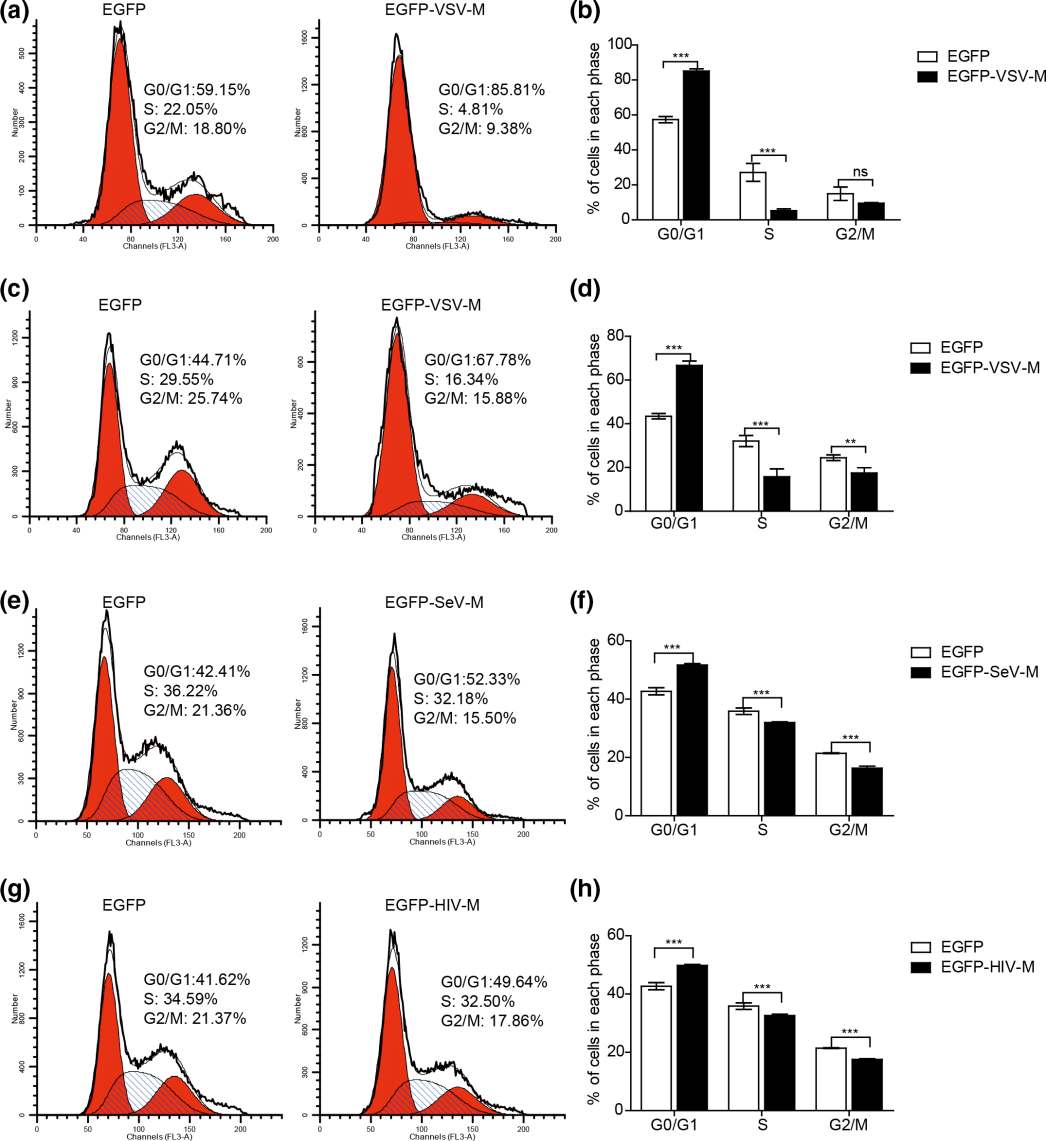
M proteins from VSV, SeV and HIV induce cell cycle blocked at G0/G1 phase. (a) A549 cells were transiently transfected with pEGFP or pEGFP-VSV-M plasmids. Then, 24 h later, the cells were synchronized at G0/G1 phase by serum starvation for 48 h, then Dulbecco's Modified Eagle Medium (DMEM) containing 10 % FBS was added to trigger cell cycle reentry, 18 h later, cells were harvested, stained with PI, and analysed the cell cycle by flow cytometry. GFP-positive cells were selected for analysis. (b) The percentage of cells in each phase of the cell cycle is shown. (c, e, f) HeLa cells were transiently transfected with pEGFP or pEGFP-VSV-M (c), pEGFP-SeV-M (e) and pEGFP-HIV-M (g) plasmids. Then, 24 h later, cells were synchronized at G0/G1 phase by serum starvation for 36 h, then cultured for 18 h in 10 % FBS-containing medium, cells were collected and cell cycle profiles were determined by flow cytometry. GFP-positive cells were selected for analysis. (d, f, h) The percentage of cells in each phase of the cell cycle is shown, pEGFP-VSV-M (c), pEGFP-SeV-M (e), pEGFP-HIV-M (g). Data correspond to the mean±sd of at least two independent experiments. **P<0.01, ***P<0.001.

Next, we investigated whether some M proteins showed interaction with SLD5 by the yeast two-hybrid assay (Fig. 1b) can directly affect host cell cycle progression. A549 cells were transiently transfected with EGFP or EGFP-VSV-M. PI and GFP double positive cells were analysed (Fig. S4b). Expression of VSV M protein induced cell cycle arrested at G0/G1 phase compared to the EGFP alone (as 85.13±0.71% of cells transfected with EGFP-VSV-M compared to 57.37±1.04 % of control cells) (Fig. 4a, b). We got similar results with HeLa cells, expression of VSV M also blocked cell cycle progression in HeLa cells as well (Fig. 4c, d). We further tested the influence of M proteins from SeV or HIV on cell cycle, and found that SeV or HIV M protein also arrested cell cycle in G0/G1 phase in HeLa cells, although their effects were not as pronounced as VSV M (Fig. 4e–h). Taken together, these data demonstrated that the M proteins interacting with SLD5, such as VSV-M, SeV-M and HIV-M, all directly induce host cell cycle arrest.

### Nuclear localization is required for VSV M to arrest cell cycle

**Fig. 5.**
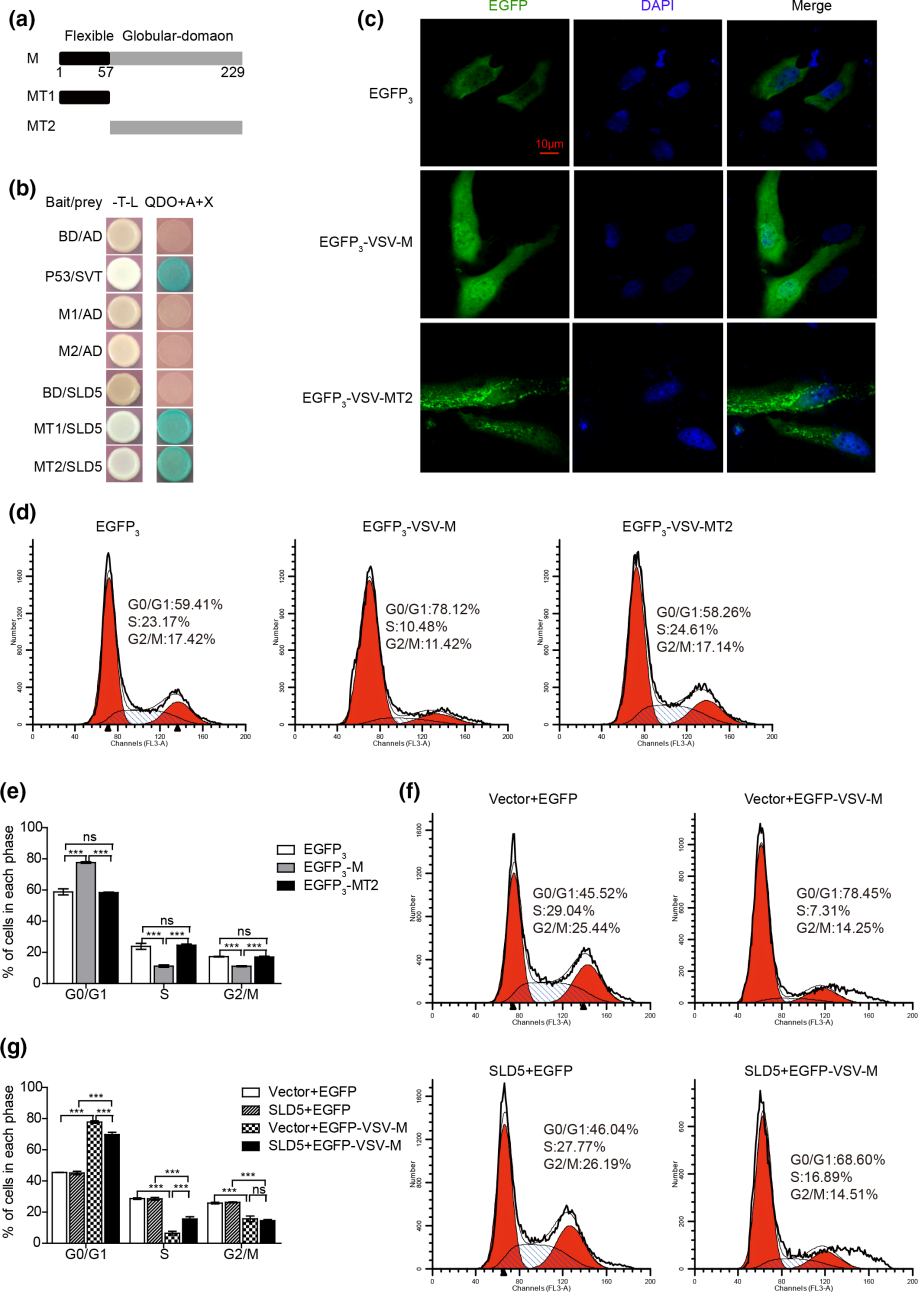
SLD5 partially rescues M-induced cell cycle arrest. (a) Schematic diagram showing the VSV M domains tested in this study. MT1 (1-57aa), MT2 (58-229aa). (b) Interaction between SLD5 and VSV M trunctions by yeast two-hybrid assay. SLD5 was fused to the pGADT7 vector, M trunctions were fused to the pGBKT7 vector. The indicated plasmids were co-transformed into yeast strain Y2HGold. Transformants were selected for growth on -T-L medum. The colonies were then transferred to -T-L (left) and QDO+A+X (right) plates. (c) Localization of the EGFP_3_-VSV-M/-MT2 constructs. HeLa cells were transfected with EGFP_3_, EGFP_3_-VSV-M or EGFP_3_-VSV-MT2 plasmids, 24 h later, cells were fixed and stained with DAPI, then analysed by confocal microscopy. Scale bars, 10 µm. (d) HeLa cells were transiently transfected with EGFP_3_, EGFP_3_-VSV-M or EGFP_3_-VSV-MT2 plasmids. Then, 24 h later, cells were serum starved for 36 h, then DMEM media containing 10 % FBS was added. Then, 18 h later, cells were harvested, stained with PI, and analysed the cell cycle by flow cytometry. GFP-positive cells were selected for analysis. (e) The percentage of cells in each phase of the cell cycle is shown. (f) HeLa cells were transfected with EGFP or EGFP-VSV-M in combination with the empty vector or SLD5, 24 h later, cells were serum starvation for 48 h, then DMEM media containing 10 % FBS was added, 18 h later, collected the cells and analysed the cell cycle by flow cytometry. GFP-positive cells were selected for analysis. (g) The percentage of cells in each phase of the cell cycle is shown. Data correspond to the mean±sd of three independent experiments. ***P<0.001.

VSV M is a 229 amino acid protein comprising of two domains, a flexible N-terminal domain (MT1, 1-57aa) and a globular C-terminal domain (MT2, 58-229aa) [32, 33] (Fig. 5a). To identify which domain is required for interaction with SLD5, each domain of M was cloned into pGBKT7 vector, then their interactions with SLD5 were detected by yeast two-hybrid assay. As shown in Fig. 5(b), both the two domains showed interaction with SLD5. VSV genome replicates in the cytoplasm [34], but M protein locates both in the nucleus and cytoplasm of VSV-infected cells [12]. VSV-M contains NLS and its nuclear localization is essential for inhibiting nucleocytoplasmic transport [35, 36]. To investigate whether the nucleus location of M protein is required for its ability to manipulate cell cycle, we generated fusion proteins that contain three tandem copies of EGFP_3_ and the full length VSV M (EGFP_3_-VSV-M) or the truncation VSV M (58-229aa, EGFP_3_-VSV-MT2). Firstly, we determined the cellular localization of EGFP_3_, EGFP_3_-VSV-M and EGFP_3_-VSV-MT2. As shown in Fig. 5(c), EGFP_3_ and EGFP_3_-VSV-MT2 were located almost exclusively cytoplasmic, while EGFP_3_-VSV-M was accumulated strongly in cell nuclei, these results were consistent with previously reported [36]. Then we transiently transfected HeLa cells with EGFP_3_, EGFP_3_-VSV-M or EGFP_3_-VSV-MT2, and analysed cell cycle. As shown in Fig. 5(d, e), expression of EGFP_3_-VSV-M significantly induced cell cycle blockade at G0/G1 phase, as approximately 77.67±0.78% of EGFP_3_-VSV-M expressing cells were at G0/G1 phase, compared with 58.82±2.94% of EGFP_3_ or 58.37±0.48% of EGFP_3_-VSV-MT2 expressing cells at G0/G1 phase, respectively. These results indicated that the nuclear localization of VSV M is required for its effect on the cell cycle.

### SLD5 partially rescues VSV-M-induced host cell G0/G1 arrest

We further determined whether SLD5 could rescue the VSV-M-induced cell cycle arrest. HeLa cells were transfected with EGFP or EGFP-VSV-M in combination with the empty vector or SLD5, followed by cell cycle analysis. As shown in Fig. 5(f, g), expression of M significantly increased the proportion of G0/G1 cells from 45.49±0.61 to 77.81±1.09% (vector+EGFP transfected cells vs. vector+EGFP-VSV-M transfected cells). Meanwhile, co-expression of SLD5 markedly decreased the VSV-M-induced cell cycle blockade (69.79±2.15% in SLD5 co-expressing cells compared to 77.81±1.09% in only M expressing cells), demonstrating that SLD5 attenuates VSV-M-induced cell cycle arrest.

### SLD5 suppresses VSV replication

**Fig. 6.**
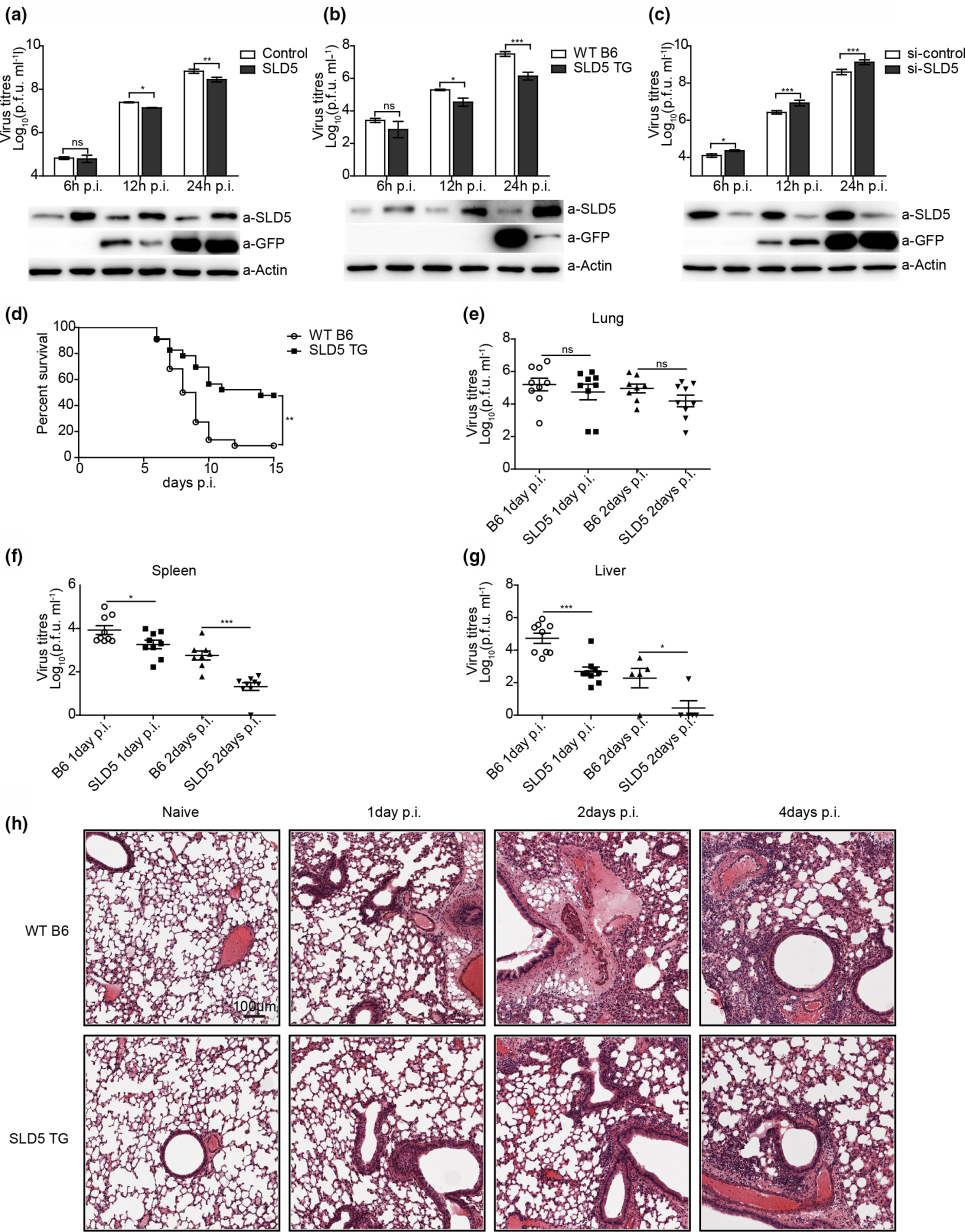
SLD5 suppresses VSV replication *in vitro* and vivo. (a) Virus replication in A549 SLD5 overexpressing cells. The SLD5 overexpressing and control A549 cells were infected with VSV-GFP virus (m.o.i.=0.01), supernatants and cells were collected at the indicated timepoints, and virus litres were determined by plaque assays on DF-1 cells. The expression level of SLD5 and GFP were detected by Western blot. (b) Virus replication in primary MEFs. Primary MEFs from WT B6 and SLD5 TG mice were infected with VSV-GFP virus (m.o.i.=0.01), supernatants and cells were collected at the indicated timepoints, and virus litres were determined by plaque assays on DF-1 cells. The expression level of SLD5 and GFP were detected by Western blot. (c) Virus replication in siRNA-treated A549 cells. Cells were transfected with siRNA 24 h before being infected with VSV-GFP virus (m.o.i.=0.01), supernatants and cells were collected at the indicated timepoints, and virus litres were determined by plaque assays on DF-1 cells. The expression level of SLD5 and GFP were detected by Western blot. (d–h) WT B6 and SLD5 TG mice were intranasally infected with 1×10^7^ p.f.u. VSV virus, the survival rate of infected mice was monitored (d), virus litres in the lungs (e), spleens (f), and livers (g) were determined by plaque assays on DF-1 cells. (h) HE staining of the lung sections at 1, 2 and 4 days p.i. Scale bars, 100 µm. Data correspond to the mean±sd of at least two independent experiments. *P<0.05, **P<0.01, ***P<0.001.

So far, our data showed that SLD5 partially rescued VSV-infection or VSV M expression-induced G0/G1 phase arrest. To investigate whether SLD5 affects viral replication, we firstly infected A549 SLD5-overexpressing cells or control cells with VSV-GFP virus, the culture supernatants were collected at 6, 12 and 24 h p.i., and titrated on DF-1 cells. As shown in Fig. 6(a), overexpression of SLD5 significantly inhibited virus replication at 12 and 24 h p.i., resulting in reduced virus litres in the supernatants, GFP expression level was also lower in SLD5-overexpressing cells than that in control cells, especially at 12 h p.i. To further determine the effect of SLD5 in VSV infection, we isolated primary mouse embryonic fibroblast cells (MEFs) from 13.5 day embryos of wild-type C57BL/6 (WT B6) and SLD5 transgenic (SLD5 TG) mice as described previously [16]. Then the cells were infected with VSV-GFP, the culture supernatants were collected at different time points after infection and titrated on DF-1 cells. The virus litres were significantly lower in the SLD5 TG MEFs than that of WT MEFs at 12 and 24 h p.i., and GFP expression level was lower in SLD5-overexpressing cells than that in control cells as well (Fig. 6b).

We next knocked down SLD5 in A549 cells by using SLD5-specific siRNA and used a scrambled siRNA (si-Control) as the negative control. After being treated with siRNA, the cells were infected with VSV-GFP, the culture supernatants were collected at different time points after infection and titrated on DF-1 cells. Western blotting confirmed the SLD5 knockdown was successful. The virus litres and the expression of GFP were both increased in SLD5 knockdown cells compared with that of control cells (Fig. 6c). Collectively, these data demonstrated that SLD5 inhibits VSV-GFP virus replication *in vitro*.

Next, we explored the physiologic role of SLD5 during VSV infection, WT B6 and SLD5 TG mice were intranasally (i.n.) infected with 1×10^7^ p.f.u. VSV virus. As shown in Fig. 6(d), SLD5 TG mice showed significantly higher survival rate than WT B6 mice after VSV infection. At 15 days post-infection (days p.i.), only 10 % of WT B6 mice survived, while about half of the SLD5 TG mice were still alive. We also examined the virus litres in various organs after infection. The virus litres in the lungs showed no significant differences between WT B6 and SLD5 TG mice at 1 and 2 days p.i. (Fig. 6e). However, SLD5 TG mice exhibited markedly decreased virus litres in the spleens and livers than that of WT B6 mice both at 1 and 2 days p.i. (Fig. 6f, g). Hematoxylin-and-eosin (HE) staining showed greater infiltration of immune cells and damage in the lungs of WT B6 in comparison to that of SLD5 TG mice after VSV infection (Fig. 6h). Thus, high level of SLD5 expression provided survival advantage for mice after VSV infection.

### SLD5 promotes antiviral innate immune responses

**Fig. 7.**
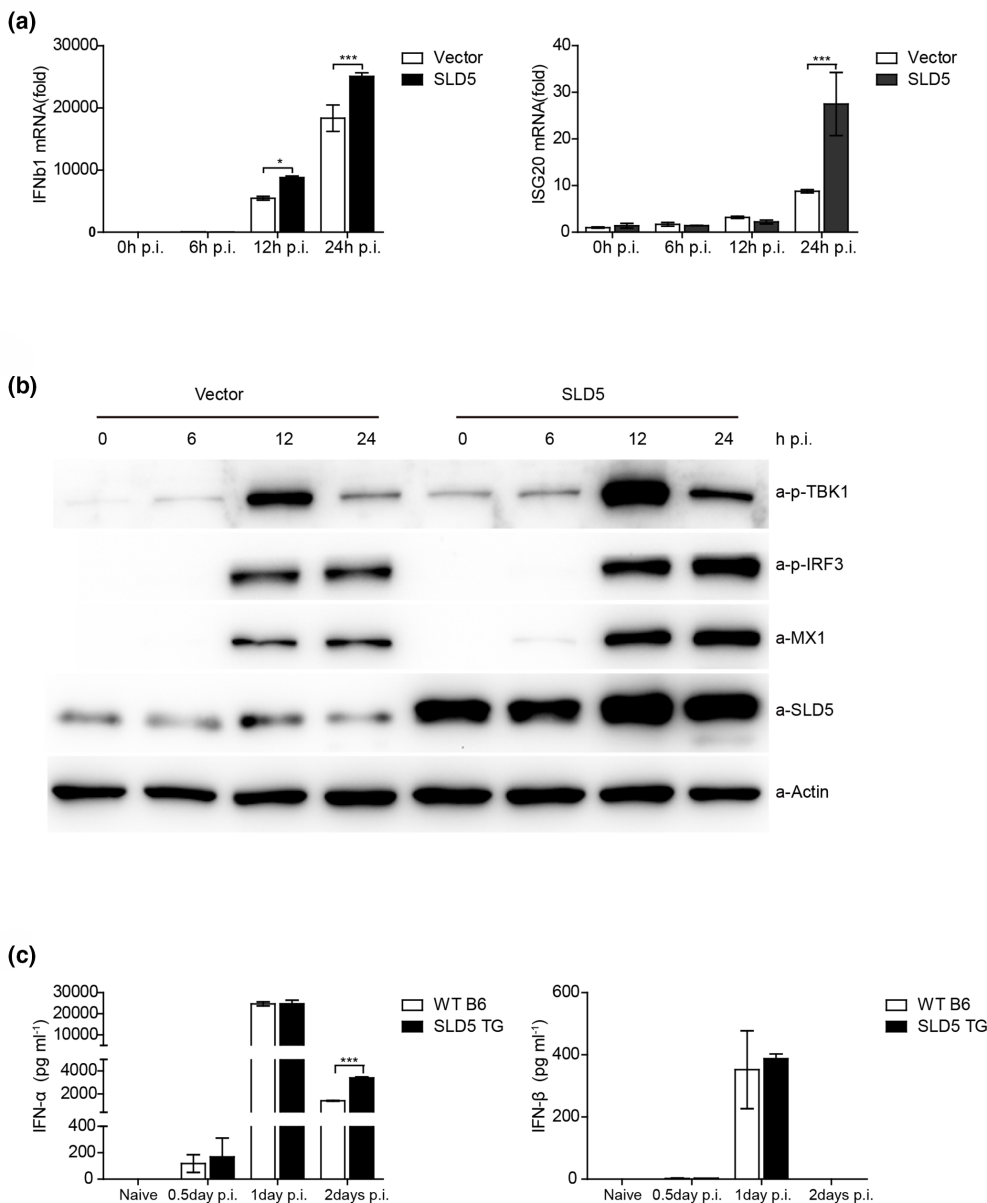
SLD5 promotes antiviral innate immune response. (a) qRT-PCR analysis of *Ifnb* and *Isg2*0 mRNA. A549 SLD5 overexpressing and control cells were infected with VSV-GFP virus (m.o.i.=1), Quantitative real-time reverse transcription PCR (qRT-PCR) was performed at indicated time points post-infection. (b) Immunoblot analysis of the indicated proteins in A549 SLD5 overexpressing and control cells infected with VSV-GFP virus (m.o.i.=1) for the indicated times. (c) WT B6 and SLD5 TG mice that were intranasally infected with 1×10^7^ p.f.u. VSV. IFN-α and IFN-β in the sera at the indicated time points post-infection were determined by ELISA. The data correspond to the mean±sd for at least two independent experiments. *P<0.05; ***P<0.001.

Previous studies have shown that G2/M cell cycle arrest strongly enhances the replication of VSV-ΔM51 (but not of wild-type VSV) via inhibition of antiviral gene expression, likely due to mitotic inhibition of transcription [37]. Since many viruses have been shown to induce G0/G1 arrest, it would be interesting to investigate whether cell cycle arrested at G0/G1 would also affect antiviral gene expression. Our previous results showed that SLD5 partially rescues VSV infection-induced host cell G0/G1 arrest, then we investigated whether SLD5 could affect type I IFNs because type I IFNs are key mediators of the host antiviral responses, and VSV is very sensitive to the type I IFN-mediated innate immune defenses [38]. Upon virus infection, activated TBK1 phosphorylates IRF3 and triggers its nuclear translocation, which ultimately promotes the production of type I IFNs and ISGs [39]. As shown in Fig. 7(a), overexpression of SLD5 increased the mRNA levels of *Ifnb1* at 12 and 24 h p.i., and *Isg20* at 24 h p.i. induced by VSV infection. Furthermore, the phosphorylation of TBK1 and IRF3, and the level of interferon-induced antiviral protein MX1 were markedly higher in SLD5-overexpressing cells than that of control cells after VSV infection, as demonstrated by Western blot in Fig. 7(b).

Next, in order to evaluate whether SLD5 affects antiviral type I IFN responses *in vivo*, we infected WT B6 and SLD5 TG mice with VSV. IFN-α and IFN-β from sera were determined by ELISA. As shown in Fig. 7(c) , the levels of IFN-α peaked on 1 days p.i. and slightly decreased at 2 days p.i., the sera IFN-α level of SLD5 TG mice was significantly higher than that of the WT B6 mice at 2 days p.i.; while the level of IFN-β peaked at 1 days p.i. and almost vanished at 2 days p.i., the sera IFN-β level of SLD5 TG mice was slightly higher than that of the WT B6 mice at 1 days pi. Collectively, these findings indicate that SLD5 promotes the type Ⅰ IFN signalling during VSV infection.

## Disscusion

Viral M protein is an integral part of the virus particle and plays numerous functions during virus assembly, budding and virion production [1, 2]. The life cycle of some viruses, such as influenza virus, occurs in the nucleus and cytoplasm [40], while the life cycle of other viruses, such as VSV, occurs only in the cytoplasm [34]. However, most M proteins from those enveloped RNA viruses contain NLS and can shuttle to the host cell nucleus (Table 1, Fig. 1a). SLD5 is a component of the GINS complex, which is essential for initiation of DNA replication and the G1/S phase transition [41]. Previously we identified that the M1 of influenza virus induces cell cycle arrest at G0/G1 phase by interacting with SLD5 [16]. In this study, we found that the M protein from several RNA viruses, including ICV, MeV, SeV, VSV, ZEBOV and HIV all interacted with SLD5. Furthermore, the M protein of VSV, SeV or HIV all induced cell cycle arrest at G0/G1 phase. SLD5 partially rescued the cell cycle arrest induced by VSV/SeV infection or VSV/SeV M expression, indicating that SLD5 might be a common target for viral M proteins to manipulate host cell cycle.

The detailed binding sites between human SLD5 and different viral M proteins in the modelled complexes were quite different (Fig. S3), which may be due to the amino acid sequences of M protein from different RNA viruses are not conserved (Fig. S2) and their structures are diverse [1]. For example, the structure of influenza virus M1 is completely α-helix [42], while the structure of VSV M is mainly β-sheet [32]. Despite major sequence and structural differences between M proteins, they all interact with SLD5, which indicates that different RNA viruses might evolve to target the highly conserved eukaryotic cell DNA replication mechanism by M protein.

VSV-M consists of two domains, a flexible N-terminal domain (MT1, 1-57aa) and a globular C-terminal domain (MT2, 58-229aa). MT1 contains the NLS sequences and is required for the nuclear localization of M protein. Consistent with previous reports, we found that the cellular localization of EGFP_3_ and EGFP_3_-VSV-MT2 were almost exclusively cytoplasmic, however EGFP_3_-VSV-M was accumulated strongly in cell nuclei. Expression of EGFP_3_-VSV-M significantly induced cell cycle blockade at G0/G1 phase, while EGFP_3_-VSV-MT2 had no effect on cell cycle. We further detected the cellular localization of MT1, as shown in Fig. S5, MT1 was widely distributed in both cytoplasm and nucleus. However, MT1 showed no effect on cell cycle as there were no differences in the proportion of cells at each cell phase between the EGFP and EGFP-VSV-MT1 expressing HeLa cells (Fig. S6). Because MT1 (1–57 aa) is short and contains the NLS sequences, even though MT1 showed interaction with SLD5 in our yeast two-hybrid assay (Fig. 5b), the interaction of MT1 with SLD5 might be weak. Also, as the predicted interactions within the interface of SLD5 and VSV M protein by the Discovery Studio 2.5 (Fig. S3), the important residues involved in hydrogen bond interactions of VSV M protein with SLD5 are all located in the MT2 domain (R101, H90, N162, G164, K213, S198) of VSV M. Thus, our results indicated that the nuclear localization of VSV M is required for its regulation of the cell cycle. Therefore, the direct interaction between M and SLD5 in the nucleus are likely to disrupt the formation of the GINS complex, resulting in cell cycle arrest.

Viruses rely on infected cells to provide resources for replicating their genome. Manipulating the cellular machinery that controls replication is a common activity of many viruses [13]. We found that VSV and SeV infection induced cell cycle arrest at G0/G1 phase in A549 and HeLa cells, and SLD5 partially rescued the cell cycle arrest. Cell cycle perturbations have been reported for VSV. One study suggested that successful cell cycle transition from G0 to G1 phase is required for VSV replication in primary T lymphocytes [43]. In contrast, another study stated that neither the cell cycle progression nor the translation control is essential for VSV replication in hepatocellular carcinoma cells [44]. In addition, it has been reported that VSV infection interferes with mitotic progression and triggers cell death in normal rat kidney cells [45]. Cell cycle arrest in G2/M phase can enhance the replication of VSV variant (VSV-ΔM51), which is more sensitive to type I IFN antiviral responses than that of wild-type VSV [37]. VSV infection arrests varied cell cycle blockage, indicating that the effect of VSV on cell cycle may be cell type specific.

VSV primarily affects ungulates and causes characteristic vesicular lesions around the mouth, nose, teats and coronary bands [46]. Many host proteins have been identified to inhibit VSV replication. It has been reported that the eukaryotic translation initiation factor 3, subunit i (eIF3i) affects the growth of VSV by interacting with its M protein [33]. Other host factors, such as the major inducible 70 kDa heat shock protein (Hsp70), promotes type Ⅰ IFN-dependent antiviral response against VSV in neurons [47]. Cancer upregulated gene 2 (CUG2), a new oncogene, confers resistance to VSV infection through STAT1-OASL2 signalling pathway [48]. Type Ⅰ IFNs have the potential to influence many aspects of virus infection, and IFN-α/β receptor-deficient mice are highly susceptible to VSV infection despite the presence of an otherwise intact immune system [49, 50].

We found that SLD5 inhibited VSV replication and boosted VSV-induced type Ⅰ IFN signalling. However, it is difficult to distinguish whether SLD5 inhibits viral replication by affecting host cell cycle or by interacting with M protein, or because of the enhanced type Ⅰ IFN responses. In order to gain some insights into this question, we firstly arrested the A549 SLD5-overexpressing cells or control cells in G0/G1 phase, and then infected with VSV. We found that there were no differences in the type I IFN responses between the two groups of cells (Fig. S7). This data indicated that SLD5 might function before the cell cycle fully arrested in G0/G1 phase. Furthermore, we infected type I IFN-deficient Vero cells, and found that there were no differences in the virus litres between SLD5-overexpressing or control Vero cells at 6 and 12 h p.i. At 24 h p.i., the virus litres were lower in the SLD5-overexpressing cells, however, the effect was quite modest (Fig. S8). Thus, our results suggested that type Ⅰ IFN responses might play an important but not solely role in the reduced viral replication in the SLD5 overexpressing cells or TG mice (Fig. 6). However, the mechanisms of how SLD5 promotes the type Ⅰ IFN pathway deserve further investigations. In addition, the inherent hydrophobic characteristics of M protein make it prone to oligomerization, which limits the research of biochemistry and structural biology [1]. It is not clear whether SLD5 interacts with monomeric or polymeric M protein. The interaction of SLD5 and M protein may affect the formation of progeny virions.

In conclusion, our study indicated that SLD5 might be a common target by some RNA viruses to manipulate host cell cycle. M proteins from several enveloped RNA viridae interact with SLD5, suggesting that different RNA viruses might evolve to target the highly conserved eukaryotic cell DNA replication mechanism by their M proteins. Therefore, our data revealed a new function of M protein from RNA viruses, and highlighted a role of the conserved cellular DNA replicating apparatus in viral infections.

### Experimental procedures

#### Virus and plaque assay

VSV-GFP and VSV (Indiana strain) viruses were kindly provided by Zhengfan Jiang (Peking University, Peking, PR China) and propagated as described previously [51]. Briefly, the virus was propagated by infection of a monolayer of DF-1 cells. Then, 24 h later, the supernatant was harvested and stored in aliquots at −80 °C. Viral litres were determined by plaque assay on DF-1 cells. In brief, DF-1 cells were incubated with culture supernatants from infected cells or homogenates of organs from infected mice at serial dilutions in serum-free DMEM for 2 h at 37 °C. Then the virus inoculums were removed by washing with PBS. The cell monolayers were overlaid with agar medium DMEM supplemented with 1% low-melting-point agarose and incubated at 37 °C for 24–48 h. The plates were fixed with 4% paraformaldehyde for 1 h, the agarose overlays were carefully removed. Staining buffer (0.1% crystal violet and 20% ethanol in water) was added to the wells and incubated for at least 10 min. The staining buffer was subsequently aspirated. The plaques were counted and the virus litres were calculated accordingly.

#### Cells

Overall, 293 T cells (ATCC, CRL-3216), A549 cells (ATCC, CCL-185), HeLa cells (ATCC, CCL-2), MEFs (isolated as previously described [16]) and DF-1 cells (kindly provided by Dr Zhengfan Jiang, Peking University, PR China) were maintained in DMEM supplemented with 10% FBS (Gibco), 2 mM l-glutamine (Hyclone), 100 µm non-essential amino acids (Hyclone), 10 mM HEPES (Sigma), 0.05 mM β-mercaptoethanol (Sigma), 100 IU ml^−1^ penicillin and 100 µg ml^−1^ streptomycin (Invitrogen). All cells were cultured at 37 °C in 5% CO_2_.

For infection, cells were infected with VSV-GFP virus at indicated m.o.i. After 1 h, cells were washed with PBS and cultured in DMEM containing 10% FBS. Cell supernatants were collected at different time points post-infection and virus litres were determined by plaque assays on DF-1 cells.

#### Constructs and antibodies

The human SLD5 gene was cloned into pCDH, pGADT7, PCI-EGFP and pcDNA4.0-SLD5-HA vectors; the M genes of ICV, MeV, SeV, HRSV, VSV, ZEBOV, HIV, EIAV and truncated VSV M were cloned into pGBKT7; M genes of VSV and HIV were cloned into pcDNA3.0-flag; M genes of VSV, SeV and HIV were cloned into pCI-EGFP; the full length and truncated VSV M were cloned into the pCI-EGFP_3_ vector.

Rabbit anti-SLD5 sera were generated as previously described [16]. Mouse anti-flag mAb (F1804) was purchased from Sigma; goat anti-HA-tag polyclonal antibody (A00168-40) was purchased from GenScript; mouse anti-β-actin (sc-47778) mAb was purchased from Santa Cruz Biotechnology; rabbit anti-phospho-IRF3 mAb (Ser386, ab76493), rabbit anti-phospho-TBK1 mAb (Ser172, ab109272) and rabbit anti-MX1 (ab95926) Abs were purchased from Abcam. Mouse anti-GFP mAb (AB1007t) was purchased from Boao Rui Jing Biotechnology; Horseradish Peroxidase (HRP)-conjugated goat anti-mouse/rabbit IgG (ZB-2305/ZB-2301) and TRITC-conjugated goat anti-mouse IgG (ZF-0313) were purchased from Zhongshan Golden Bridge Biotechnology. FITC-conjugated rabbit anti-goat IgG antibody (bs-0294R) was purchased from Bioss.

#### Cell cycle analysis

A549 and HeLa cells were firstly synchronized in the G0/G1 phase with medium containing no serum for 48 h and 36 h, respectively, when needed. Then DMEM containing 10 % FBS was added to trigger cell cycle reentry. Then, 18 h later, the cells were trypsinized and collected, washed with PBS, then fixed in cold 70% ethanol overnight at 4 °C. After fixation, the cells were washed once with PBS and resuspended in staining buffer (50 g ml^−1^ PI, 20 g ml^−1^ RNase A, 0.1% Triton X-100 in PBS) for 20 min at room temperature. PI stained cells were analysed using a FACS Calibur flow cytometer (BD Biosciences). For GFP transfected cells, PI and GFP double positive cells were analysed with ModFit LT version 2.0. At least 20 000 cells were counted for each sample. For infection experiments, synchronous or asynchronous cells were directly infected with VSV-GFP or SeV at indicated m.o.i. After 1 h adsorption, cells were washed with PBS and cultured in DMEM containing 10 % FBS to trigger cell cycle reentry. At 18 h p.i. (for synchronized) or 24 h p.i. (for asynchronized), the cells were harvested and subjected to cell cycle analysis as described above and previously [16].

#### Transfection

For plasmids, transfection was performed using Lipofectamine 2000 (Invitrogen) according to the manufacturer’s instructions.

For siRNA transfection, A549 cells were transfected with siRNA using Lipofectamine RNAiMAX (Invitrogen). Individual siRNA targeting human SLD5 and a scrambled siRNA were used. The sequence used for siRNA was as follow: si-SLD5 (5′-GCCTGAGATTGTAGAATGT-3′). All siRNA were purchased from Guangzhou RiboBio.

#### Lentivirus production and lentiviral transduction

The lentivirus system was purchased from System Biosciences, LLC and was performed as previously described [16]. Briefly, lentiviruses were produced by transfection of 293 T cells with either pCDH-SLD5 or the empty vector PCDH in combination with envelope and packaging plasmids. 6 h post-transfection, medium was changed to fresh complete DMEM. Then, 48 h later, the virus-containing supernatant was collected from the cells and passed through a 0.45 µm filter. Target cells were infected with virus-containing medium supplemented with 8 µg ml^−1^ polybrene (YEASEN). Next, 24 h after infection, the virus-containing medium was removed and fresh growth medium was added. Then, 24 h later, 1 µg ml^−1^ puromycin (Amresco) was added to the cell culture medium for selection of transduced cells.

#### Yeast two-hybrid assay

The yeast two-hybrid experiment was performed according to the matchmaker yeast two-hybrid system (Clontech) as previously described [16]. Briefly, the M genes of ICV, MeV, SeV, HRSV, VSV, ZEBOV, HIV, EIAV and truncated VSV M coding sequence were cloned into pGBKT7 and used as bait. The human SLD5 was cloned into pGADT7 vector and used as prey. Yeast strain Y2HGold was co-transformed with the pGBKT7-M and the prey plasmid pGADT7-SLD5. Transformants were selected on plates lacking tryptophan and leucine (-T-L), followed by further selection with high stringency quadruple dropout medium lacking tryptophan, leucine, adenine, histidine and supplemented with AbA and X-α-gal (QDO+A+X). Co-transformation of pGADT7 and pGBKT7 into Y2H Gold served as a negative control. Co-transformation of pGADT7-T-antigen and pGBKT7-p53 into Y2H Gold served as a positive control. Positive protein–protein interactions result in blue colonies in the QDO/X/A plates.

#### Co-immunoprecipitation

Co-immunoprecipitation and Western blot were conducted as previously described [16]. Transfected cells were lysed in lysis buffer (Beyotime) and cleared by centrifugation. Cleared cell lysates were incubated with anti-flag M2 affinity beads (Sigma) for 2 h. Following immunoprecipitation, the beads were washed four times with lysis buffer, imunoprecipitated samples were denatured by boiling and then resolved by SDS-PAGE and analysed by immunoblotting using indicated antibody.

#### Immunofluorescence staining

HeLa cells on glass bottom cell culture dishes were washed once with PBS and fixed with 4 % paraformaldehyde for 10 min at room temperature (RT). After washing once with PBS, cells were permeabilized with 0.1 % Triton X-100 for 10 min. After washing once with PBS, cells were blocked in PBS containing 5 % bovine serum albumin (BSA) for 1 h at RT and incubated with mouse anti-flag and goat anti-HA antibodies in PBS containing 0.1 % BSA for 2 h at RT. After three washes, cells were incubated with TRITC- and FITC-conjugated secondary antibodies for 1 h at RT and then with DAPI (BioRoYee) for 5 min. The glass dishes were washed and mounted using mounting medium (ab103746, Abcam). Images of the cells were observed with a Leica SP8 confocal microscope.

#### Mice and infection

WT B6 mice were purchased from Vital River, China. SLD5 Transgenic C57BL/6 (SLD5 TG) mice were described previously [16]. All mice were housed in an animal facility under specific pathogen-free conditions. For infection experiments, age and sex-matched B6 and SLD5 TG mice (8–10 weeks of age) were intraperitoneally anesthetized with trichloroacetaldehyde hydrate (375 mg kg^−1^ body weight) and inoculated intranasally with 1×10^7^ p.f.u. VSV virus. Organs from inoculated mice were homogenized in PBS and determined viral load by plaque assay on DF-1 cells.

#### Lung histology

Lungs from mock infected or VSV-infected mice were dissected, fixed in 4% paraformaldehyde, embedded into paraffin, sectioned, stained with hematoxylin and eosin solution as described previously [16], then examined by microscopy Leica CS2 for histological changes.

#### QRT-PCR

Total RNA was isolated from cells using Trizol reagent (Invitrogen) and then was reserve-transcribed with a first strand cDNA synthesis kit (Thermo). Expression of the indicated genes were analysed by qRT-PCR amplified using SYBR Green (Cwbio). Data were represented as the relative abundance of the indicated mRNA normalized to that of GAPDH. Primer sequences for qRT-PCR assays are as follows:

GAPDH: 5′-agccacatcgctcagacac-3′ (forward);

5′-gcccaatacgaccaaatcc-3′ (reverse).

IFNB1: 5′-ctttgctattttcagacaagattca-3′ (forward);

5′-gccaggaggttctcaacaat-3′ (reverse).

ISG20: 5′-cacccctcagcacatggt-3′ (forward);

5′-tggaagtcgtgcttcaggt-3′ (reverse).

#### ELISA

The concentration of IFN-α and IFN-β proteins in the mice sera after VSV infection were determined by the ELISA kit (740 625, Biolegend) according to the manufacturer’s instructions.

#### Statistical analysis

Unless indicated otherwise, all experiments were repeated at least three times. Statistics were analysed using two-tailed Student’s *t*-test or two-way ANOVA test. Data are presented as the means and standard deviation of the means (sd). *P* values<0.05 were considered statistically significant.

## Supplementary Data

Supplementary material 1
