# Supplementary material for: Multiple RNA virus matrix proteins interact with SLD5 to manipulate host cell cycle
Source: J Gen Virol. 2021 Dec 9;102(12):001697. doi: 10.1099/jgv.0.001697 (PMC8744269; doi:10.1099/jgv.0.001697)
Supplement: Supplementary material 1 [file jgv-102-1697-s001.pdf]

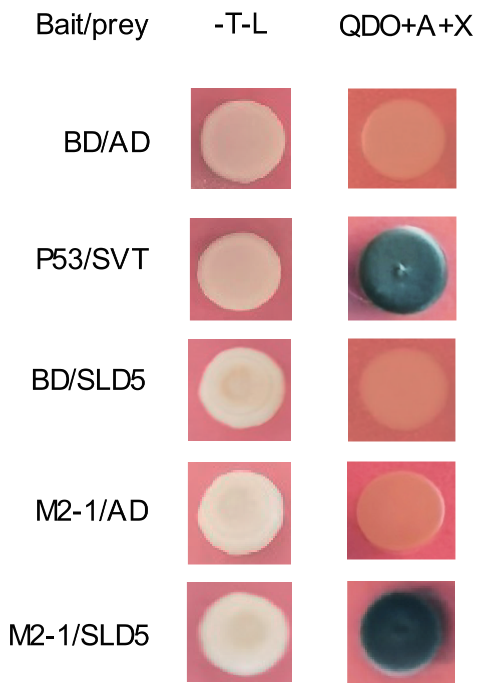

Fig S1. M2-1 protein of HRSV interacts with SLD5. Interaction between M2-1 protein and SLD5 by yeast two-hybrid assay. SLD5 was fused to the pGADT7 vector, M2-1 from HRSV was fused to the pGBKT7 vector. The indicated plasmids were co-transformed into yeast strain Y2HGold. Transformants were selected for growth on -T-L medium. The colonies were then transferred to -T-L (left) and QDO+A+X (right) plates.

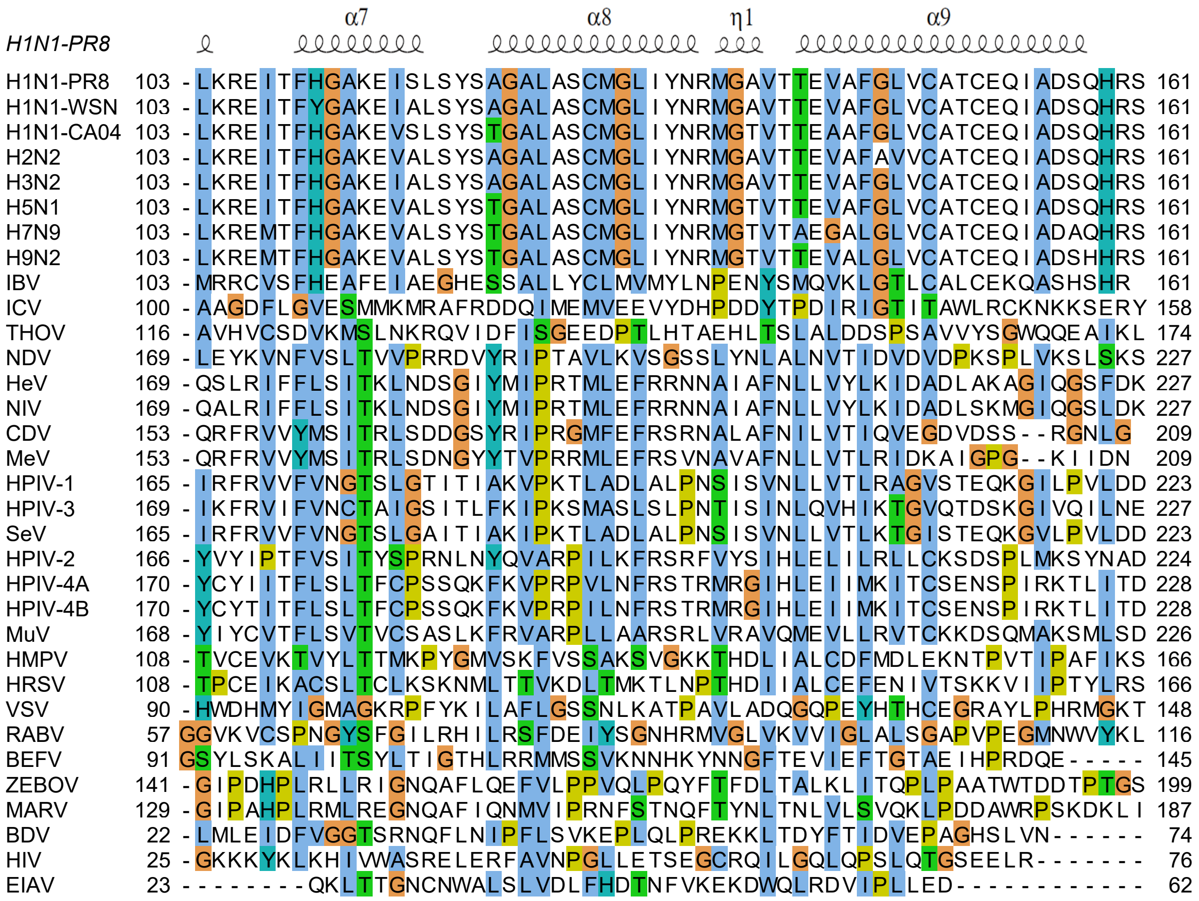

Fig S2. Sequence alignment of M proteins from the RNA viruses listed in Table 1. Sequences were aligned by Clustal W and edited by Jalview.

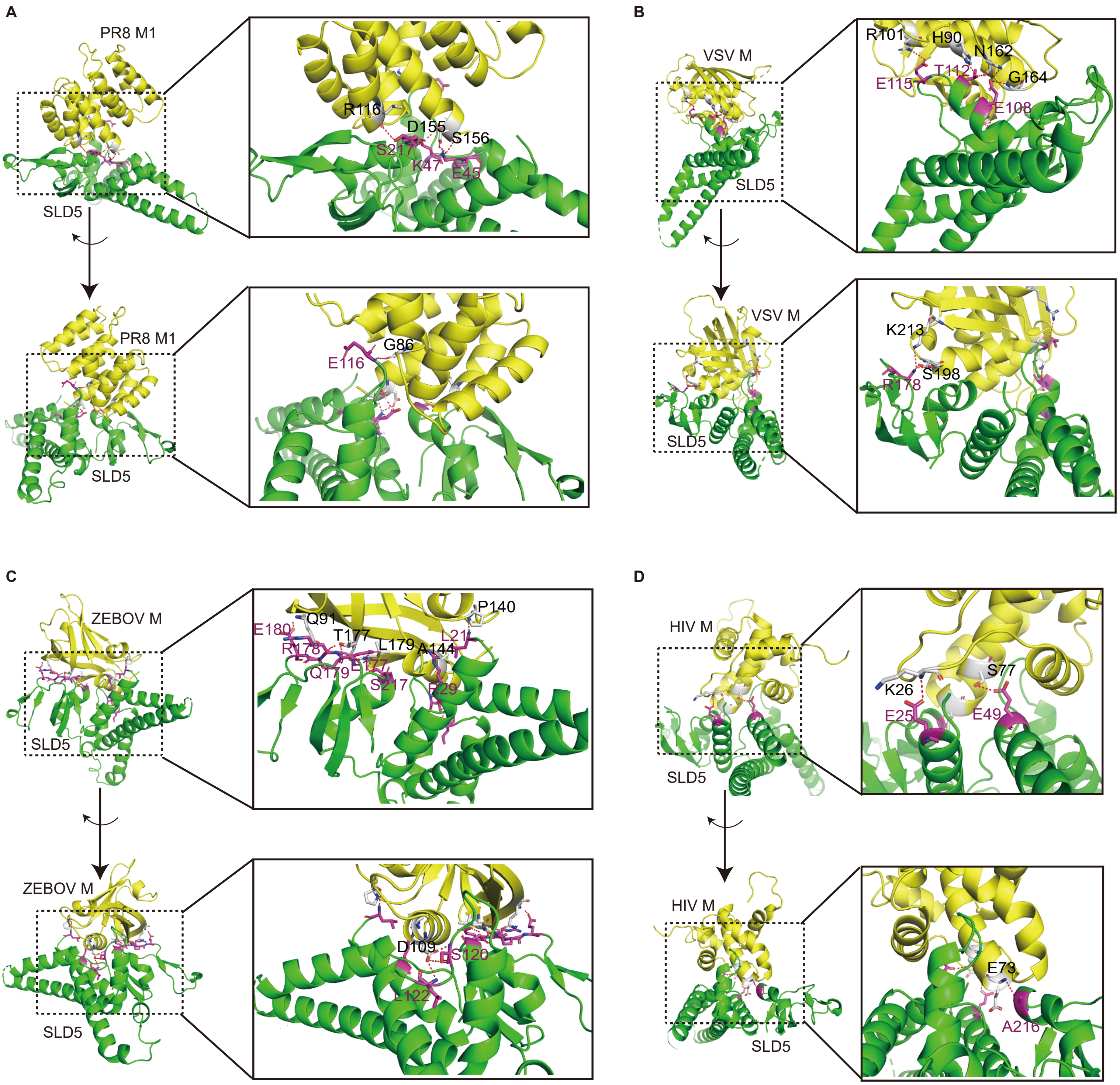

Fig S3. Detailed interactions within the interface of human SLD5 and M of PR8/VSV/ZEBOV/HIV modeled complex. SLD5 (PDB 2E9X) was defined as receptor, PR8 M1 (PDB 1EA3), VSV M (PDB 1LG7), ZEBOV M (PDB 4LDM) or HIV M (PDB 1TAM) were defined as ligand. Ligands were docked into receptor by Discovery Studio 2.5. SLD5 and M were shown as cartoon representations in green and yellow, respectively. Residues involved in interaction in SLD5 and M are colored in purple and gray. Hydrogen bonds are shown as dash lines. (A) The important residues involved in hydrogen bond interactions between residues (S217, K47, E45, E116) of SLD5 and residues (R116, D155, S156, G86) of PR8 M1. (B) The important residues involved in hydrogen bond interactions between residues (E115,T112, E108, R178) of SLD5 and residues (R101, H90, N162, G164, K213, S198) of VSV M. (C) The important residues involved in hydrogen bond interactions between residues (E180, R178, Q179, E177, S217, R29, L21, L122, S120) of SLD5 and residues (Q91, T177, L179, A144, P140, D109) of ZEBOV M. (D) The important residues involved in hydrogen bond interactions between residues (E25, E49, A216) of SLD5 and residues (K26, S77, E73) of HIV M.

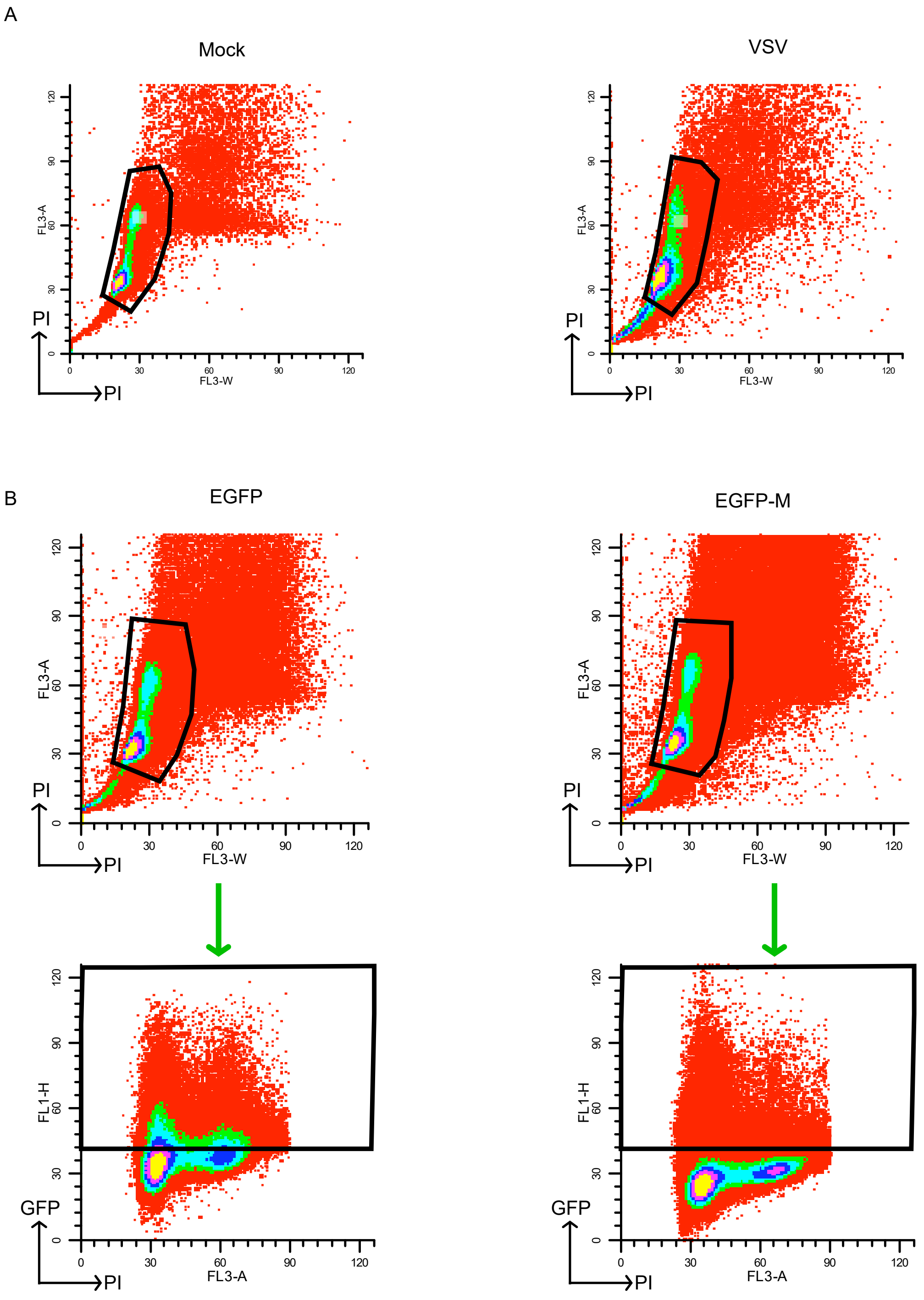

Fig S4. Interpolated histograms of some of FACS results. For non-transfected cells, PI positive cells were analysed using a FACS Calibur flow cytometer (BD Biosciences) with ModFit LT version 2.0. For GFP transfected cells, PI and GFP double positive cells were anlysed. At least 20,000 cells were counted for each sample. (A) Related to Fig 2A. (B) Related to Fig 4B.

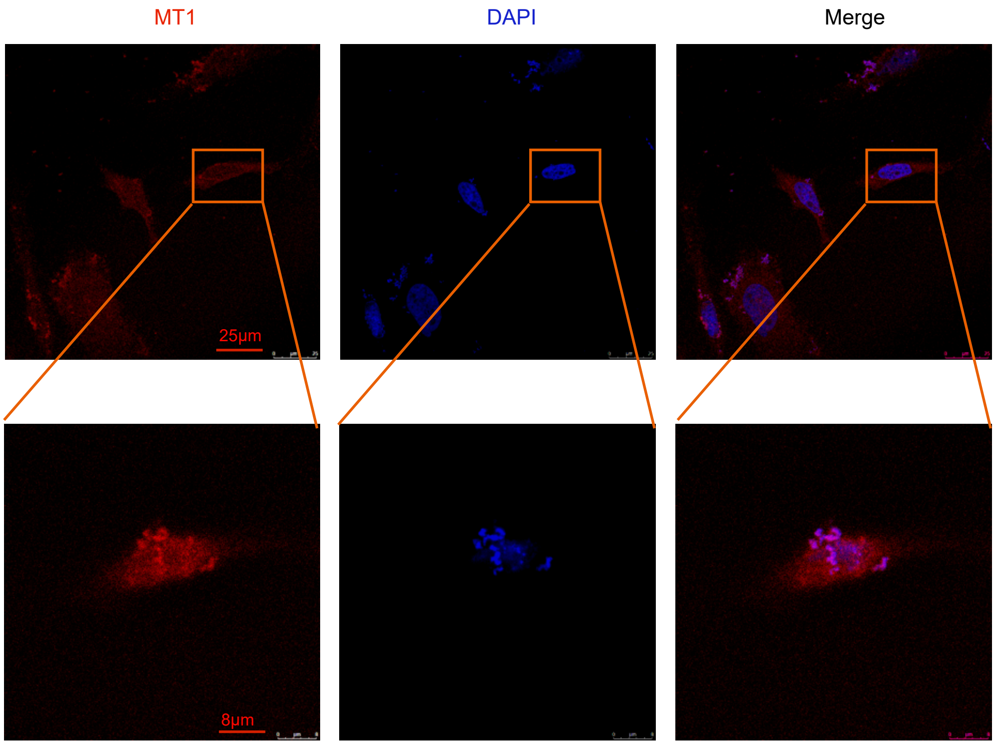

Fig S5. Localization of MT1. HeLa cells were transfected with pcDNA3.0-MT1-flag plasmid, 24h later, cells were fixed with 4% paraformaldehyde, permeabilized with 0.1% Triton X-100, stained with anti-flag antibody, followed by tetramethylrhodamine (TRITC)-conjugated secondary antibody, then with DAPI before confocal microscopy. Representative micrographs with scale bar representing 8μm and 25μm.

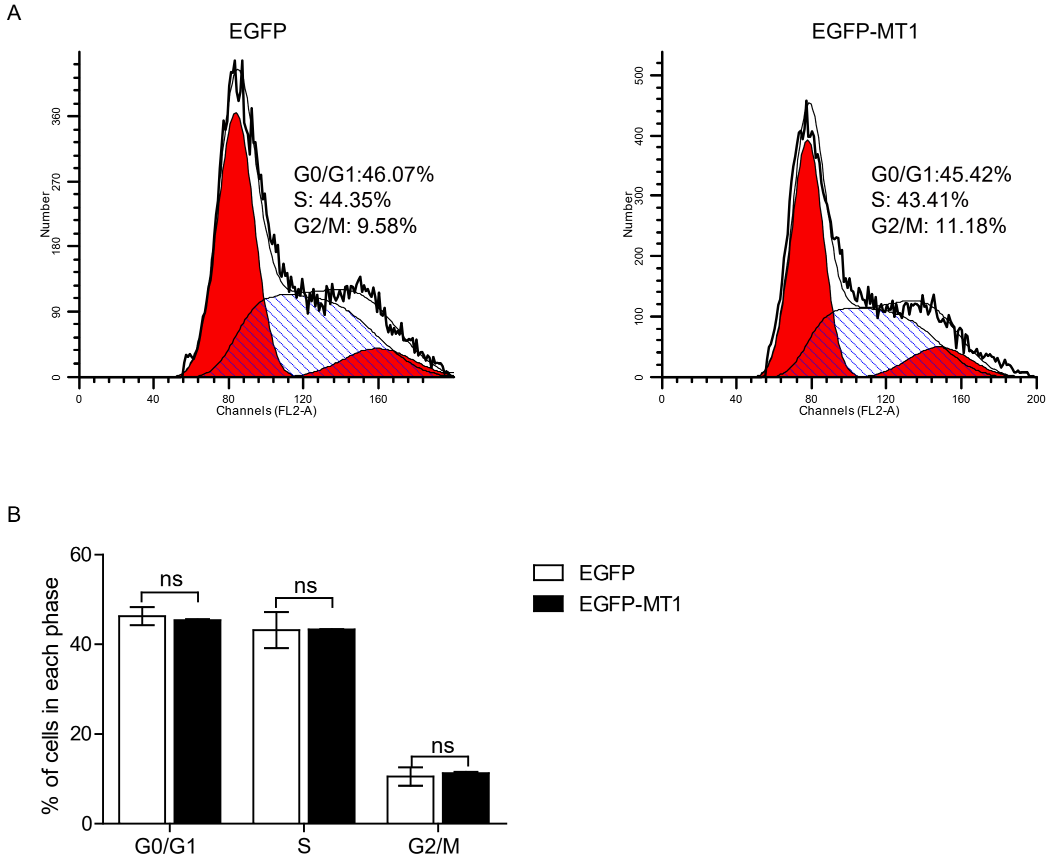

Fig S6. Effect of MT1 on host cell cycle. (A) HeLa cells were transfected with EGFP or EGFP-VSV-MT1, 24h later, cells were synchronized in G0/G1 phase by serum starvation for 48h, then DMEM media containing 10% FBS was added. 18h later, the cells were harvested, stained with PI and analyzed for cell cycle profiles by flow cytometry. GFP-positive cells were selected for analysis. (B) The histograms displaying the cell cycle distribution were analyzed by the ModFit LT program. Data correspond to the mean ± SD of two independent experiments.

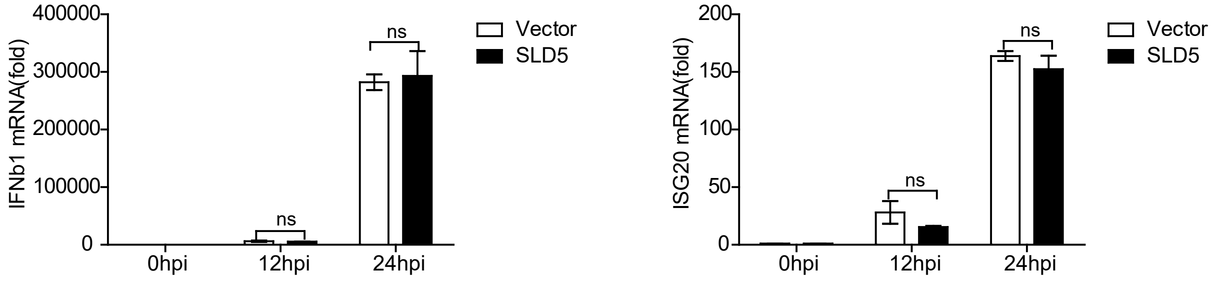

Fig S7. qPCR analysis of *Ifnb* and *Isg20* mRNA. A549 SLD5 overexpressing and control cells were firstly synchronized in the G0/G1 phase with medium containing no serum for 48h, then were untreated or infected with VSV-GFP virus (MOI=1) for indicated times before qPCR analysis.

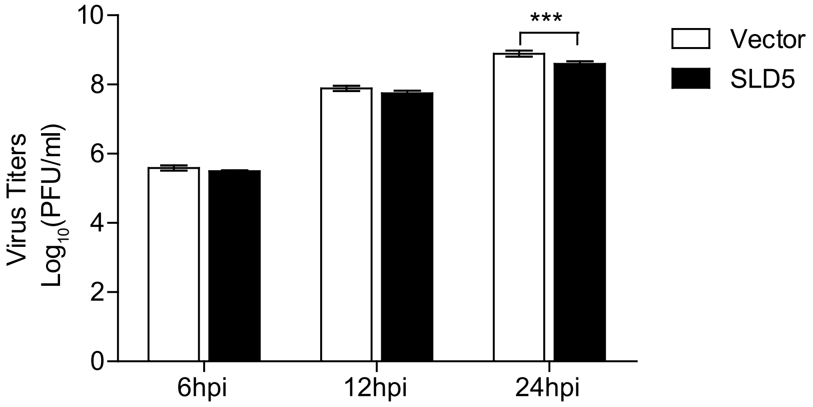

Fig S8. Virus replication in Vero SLD5 overexpressing cells. The SLD5 overexpressing and control Vero cells were infected with VSV-GFP virus (MOI=0.01), supernatants were collected at the indicated timepoints, and virus titers were determined by plaque assays on DF-1 cells. Data correspond to the mean ± SD of two independent experiments. ***p < 0.001.
